# Manufacture and characterization of a novel dairy-free quinoa yogurt fermented by modified commercial starter with *Weissella confusa*

**DOI:** 10.1016/j.fochx.2023.100823

**Published:** 2023-08-06

**Authors:** Yongyong Liu, Kai Huang, Yu Zhang, Hongwei Cao, Da-ke Luo, Cuiping Yi, Xiao Guan

**Affiliations:** a School of Health Science and Engineering, University of Shanghai for Science and Technology, Shanghai, PR China; b National Grain Industry (Urban Grain and Oil Security) Technology Innovation Center, Shanghai, PR China; c Liuyanghe Group Co., Ltd., Hunan, PR China; d School of Chemistry and Food Engineering, Changsha University of Science and Technology, Hunan, PR China

**Keywords:** Non-dairy yogurt, Quinoa, *Weissella confusa*, Fermentation, Digestion

## Abstract

Non-dairy yogurt is increasingly thought to be healthy food. However, no suitable starters limit its development. This study aimed to develop a novel and functional quinoa yogurt with a modified commercial starter. Compared with the other lactic acid bacteria (LAB), *Weissella confusa* showed a better fermentation performance of quinoa utilization. The synergistic effect of *W. confusa* and the commercial starter promoted the growth of LAB. It increased the fermentation rate of quinoa yogurt, further improving its texture, rheological properties, and storage stability. The modified starter significantly increased the nutritional qualities of the quinoa yogurt, including polyphenol content, antioxidant activity, digestive enzyme inhibition, and reduced postprandial blood glucose ability. Additionally, the modified starter enhanced the digestibility and bioaccessibility of polyphenols, protein, and fat in fermented quinoa yogurt. Overall, the commercial starter with *W. confusa* showed great potential for possible application in quinoa yogurt development*.*

## Highlights

• A novel and functional quinoa yogurt was developed with a modified starter.
• *Weissella confusa* showed a better fermentation performance of quinoa utilization.
• Synergistic fermentation improved texture and storage stability of quinoa yogurt.
• The modified starter increased the nutritional qualities of quinoa yogurt.
• Developed quinoa yogurt presented advantages in nutrients digestion.

## Graphical abstract

**Figure:**
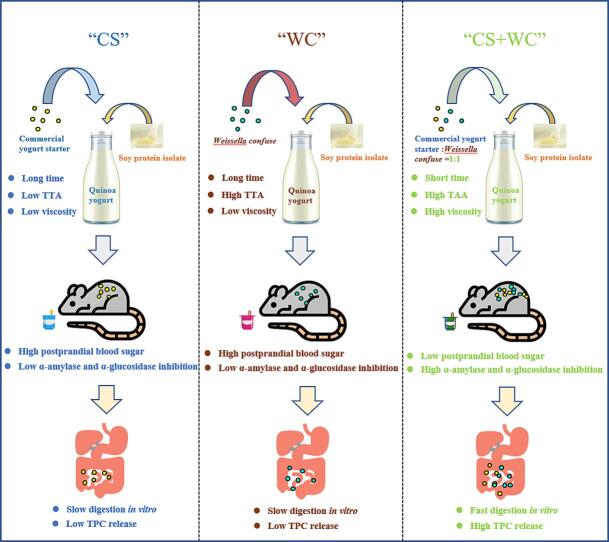

## 1 Introduction

Plant-based foods, including fruits, vegetables, seeds, beans, and their derivatives, are important components of a healthy diet. Their sufficient regular consumption could help prevent certain major diseases such as cancer and cardiovascular diseases (Bilal Shah et al., 2018). Plant-based yogurt primarily refers to products fermented from plant-based foods, which have been found to have higher nutritional value, including potential antimicrobial effects, reducing the risk of cardiovascular and gastrointestinal diseases with improved physiological functions, decreasing the risk of low bone mass and high levels of antioxidants with free radical scavenging properties (Anuyahong et al., 2020). The global plant-based yogurt market was valued at USD 1.6 billion in 2019 and is projected to grow at an annual growth rate of nearly 20% from 2020 to 2027 (Huang et al., 2022). Although plant-based yogurt products have broad market prospects, there are still problems, such as poor product taste, unstable fermentation systems, fermented strains that still need to be screened, and poor storage stability (Montemurro et al., 2021).

As a potential non-dairy production substrate, quinoa (*Chenopodium quinoa Willd*) is the only single plant recognized by the United Nations Food and Agriculture Organization (FAO) to meet the basic nutritional needs of the human body and is officially recommended as the most suitable human perfection 'Whole Nutritious Foods' (Silva et al., 2020). Quinoa grain has an excellent nutritional profile, starch (32–60%), protein (10–18%) and fat (4.4% to 8.8%), while the ashes, formed mainly from potassium and phosphorus, constitute 2.4% to 3.7% and the fiber ranges from 1.1% to 13.4% (Hussain et al., 2021). Quinoa also contains compounds like polyphenols and flavonoids. It has been reported that quinoa can be a particularly interesting resource for medical researchers, as it has a powerful natural antioxidant property (Mohamed Ahmed et al., 2021). In addition, quinoa has been reported to benefit people prone to osteoporosis, anemia, diabetes, dyslipidemia, obesity, and celiac disease (Vega-Gálvez et al., 2010).

Fermentation is a common method for improving the quality and functional properties of quinoa products. Studies have found that lactobacillus fermentation can improve quinoa's protein digestibility, texture, and sensory properties (Boeck et al., 2021). During fermentation, microbial enzymes act on phytochemicals to produce new derived compounds that have an impact on the aroma and function of fermented yogurt (Ruiz Rodríguez et al., 2021). Fermentation can also release active substances in plants, improving the nutritional function of the matrix. Plant-based yogurt is an attractive alternative for vegetarians, lactose intolerants, and people with milk allergies. Therefore, using quinoa fermentation to prepare quinoa yogurt may be an effective strategy to improve the nutrition and function of plant-based foods. Now, most studies focus on adding quinoa to milk fermentation to explore the effect of quinoa addition on ordinary yogurt (Abd-Rabou et al., 2020, Mabrouk and Effat, 2020). However, these studies all used milk as the main fermentation substrate to prepare quinoa yogurt, and quinoa was only added to yogurt in a small amount as an additive, which was not a true plant-based yogurt. There have been studies using pure quinoa to prepare high-quality quinoa yogurt to explore the nutritional value and functional properties of quinoa yogurt. But focus on exploring the effects of existing LAB strains on quinoa yogurt (Joy Ujiroghene et al., 2019).

Our previous research on fermenting quinoa yogurt with commercial yogurt starter showed that commercial yogurt starter is unsuitable for quinoa substrate and has problems such as low fermentation and long fermentation time (Huang et al., 2022). Due to commonly used probiotics being isolated from dairy sources, these strains may not have favorable conditions to grow, reproduce, and remain viable in vegan milk due to low nutrient availability, anti-nutritional factors, adverse pH, and lack of buffering capacity. The industry hopes that LAB fermented yogurt can shorten the fermentation completion time as much as possible, which is determined by the benefits of increased yield and reduced microbial contamination. Hence, a shorter fermentation time is beneficial in the industry. Therefore, this study aimed to screen out LAB suitable for the fermentation of quinoa yogurt, improve the commercial starter, and supplement soy protein to improve the fermentation degree and shorten the fermentation time of quinoa yogurt. In addition, the effect of the modified starter on the physicochemical, texture, functional, and digestive properties of quinoa yogurt was evaluated.

## 2 Materials and methods

### 2.1 Raw material

Quinoa (planted in Shanxi, China, and harvested in 2021) was purchased from Shanxi JiaQi Agricultural High-Tech Ltd. (Shanxi, China). The commercial yogurt starter was purchased from Angel Yeast Co., Ltd. (Jiangsu, China). Soy protein isolates and whey protein were purchased from Shanghai McLean Biochemical Technology Co., Ltd. (Shanghai, China).

*W. confusa, Ls.mesenteroides, Weissella halotolerans, Pediococcus pentosaseus, Sp.thermophilus, Lactobacillus reuteri, Lactobacillus plantarum, Lactobacillus bulgaricus* was obtained from Beijing Biological Conservation Center., Ltd. (Beijing, China). MRS broth was inoculated with 8 cultures of LAB and commercial starter cultures separately and grown overnight at 36 °C before use.

### 2.2 Quinoa yogurt preparation

Soak the white quinoa at 4 °C for 2 h and cook for 30 min. Quinoa was ground at a solid-liquid ratio of 1:7 (w/v) to water (Huang et al., 2022). In order to save the cost of industrial production, we use the hydrolysis quinoa solution to convert starch into sugar as a carbon source for fermentation strains. Enzymatic hydrolysis with thermostable α-amylase (3 mL/1 kg quinoa) and saccharification with saccharification enzyme (2 mL/1 kg quinoa). First, comparisons of Lactobacillus strains were performed without added protein, and the inoculum amount was guaranteed to be the same. Then the protein was screened. The slurry was divided into three groups. The first group and the second group were added with soy protein isolate and whey protein (3 g/100 mL of slurry), respectively, and the third group served as a blank control, stirred evenly, sterilized at 90 °C for 20 min, and cooled to 42 °C. Finally, the quinoa slurry with added soy protein was inoculated with commercial starter, *W. confusa,* and modified starter (commercial starter and *W. confusa* at 1:1), respectively. Different groups were inoculated with the same number of bacteria (1×10^8^ CFU/100 mL yogurt) and fermented at 42 °C for 8 h. The yogurt samples were cooled and stored at 4 °C. Analysis of yogurt after refrigeration for 1, 7, 14, and 21 days.

### 2.3 Physicochemical analysis, microbiological analysis, and color measurement

The assessment of pH, titratable acid (TTA), proximate composition, Water holding capacity (WHC), total lactic acid bacteria count (TLC), and color was performed according to the method of Huang et al. (2022).

### 2.4 Texture analysis and microscopic observation

Texture analysis was performed with a texture meter model TA-XT Plus (Stable Micro System Ltd., Surrey, UK). The assay method was carried out according to the previous protocol (Huang et al., 2022).

The scanning electron microscope (SEM) method was developed from a former one with modification (Fu et al., 2018). At least six regions were scanned for every sample, and the image with optimal contrast representing the sample’s microstructure was selected. Further treatment of images was avoided.

### 2.5 Rheological measurement

The rheological properties of the yogurt were determined by a rheometer (Discovery HR-3, TA Instruments, USA). Viscosity and stress tests are performed at 25 °C with a set shear rate in the range of 0.01 to 100 rad/s.

### 2.6 Total phenolic content and antioxidant activity analysis

Mix yogurt samples with 70% methanol (containing 0.1% hydrochloric acid) 1:1. The total phenolic content (TPC), ferric reducing antioxidant power (FRAP), DPPH radical scavenging activity, ABTS^*+^ radical scavenging activity, and Hydroxyl radical scavenging activity were determined as the method reported by Anuyahong et al. (2020) and Hwang et al. (2021). Results are expressed as mg VC/100 g Yogurt.

### 2.7 Liquid chromatography analysis of phenolic compounds

Analysis of polyphenols used previous methods with modifications (Ding et al., 2019). Unfermented samples and yogurt were centrifuged, filtered, and used as test samples. Identification of phenolic compounds in quinoa yogurt using liquid chromatography (LC). Column C18, 4.6 mm × 250 mm, 5 μm, detection wavelength 280 nm.

### 2.8 α-amylase and α-glucosidase inhibition capacity analysis

The α-amylase and α-glucosidase inhibition activity was assayed with the method reported by Hwang et al. (2021) with some modifications. Mix 50 μL of methanol extract with α-amylase and α-glucosidase solutions, add sufficient reaction substrates (starch and *p*-nitrophenyl-α-d-glucopyranoside), and determine the absorbance at 540 nm and 405 nm, respectively.

Digestive enzyme inhibition activities were calculated as follows: $Inhibition\;\left(\%\right)=\left[1-\left(A1-A2\right)/\left(A3-A4\right)\right]\times 100$

Where A1, A2, A3, and A4 represented the absorbance of the mixture containing all reagents, no substrate, no extract, and no enzyme, respectively.

### 2.9 Liquid chromatography analysis of organic acid

The analysis of organic acids uses the previous method and has been improved (Chen et al., 2021). Briefly, after centrifuging the yogurt, it was filtered through a 0.45-µm filter as the test product. Identification of organic acids in quinoa yogurt using liquid chromatography (LC). Column C18, 4.6 mm × 250 mm, 5 μm. The column temperature was 40 °C, the injection volume was 20 μL, and the detection wavelength was 210 nm.

### 2.10 Determination of postprandial blood glucose in mice

Mice fasted for 18 h before yogurt feeding, and the environment was kept at 25 ± 2 °C with 12-hour day-night alternation. The fermented yogurt that had been refrigerated for 1 day was administered by gavage, 0.1 mL/10 g of mouse body weight, and blood was collected from the tip of the tail, and the glycemic index of the mice was measured every half hour for 2 h (Y. Xu et al., 2023).

### 2.11 Simulation of gastrointestinal digestion in vitro

Unfermented extract and quinoa yogurt were used to simulate in vitro gastrointestinal digestion. The changes in the different components during the simulated in vitro digestion were determined and characterized.

#### 2.11.1 Experimental design for gastrointestinal digestion

Different types of quinoa yogurt were mixed with gastric juice containing pepsin, the pH of the digestive fluid was adjusted to 2.0 with HCl, the temperature was 37 °C, and after digestion for 120 min, the pH was adjusted to 7.4 with NaOH, and intestinal digestion solution with bile salts and pancreatic enzymes added (Silva do Nascimento et al., 2021). pH and temperature were kept constant during digestion, and samples were stored at −20 °C after 120 min of digestion.

#### 2.11.2 Determination of the content of different components of yogurt

The determination method of total polyphenol content (TPC) is the same as *2.6. Total phenolic content and antioxidant activity analysis.* The degree of protein hydrolysis (DH) was determined by the method of OPA (Ketnawa et al., 2020). The standard curve was drawn with an l-serine standard solution, and the undigested sample was used as the control group to calculate the degree of protein hydrolysis. The total sugar content (TSC) and reducing sugar content (RSC) were determined by the phenol-sulfuric acid and DNS methods, respectively. The standard curve was drawn with the standard glucose solution, and the undigested sample was used as the control group to calculate the TSC and RSC. The free fatty acid (FFA) content was detected using the free fatty acid kit from the raw materials (Ketnawa et al., 2020). The experimental steps were carried out according to the operation steps of the kit. The standard curve was drawn with the standard substance of palmitic acid, and the free fatty acid content in the sample was calculated according to the standard curve.

#### 2.11.3 Characterization of microstructure

The microstructure of the samples was analyzed by confocal laser scanning microscopy (CLSM). The methods of staining and determination were carried out as before (Huang et al., 2021). Samples were stained with 0.1% w/v Nile red, 0.01% v/v optical brightener, 0.025% w/v rhodamine, and 0.25% w/v fluorescein isothiocyanate (FITC). The concentration of each dye in the final sample was approximately 0.01 μg dye/mL. Immediately after mixing, 50 μL of the stained sample was dropped onto a glass slide, covered with a coverslip, and observed upside down. Images were taken with a 20× objective and a CLSM multiphoton system. The excitation/emission wavelengths are 488/621 nm for Nile Red, 488/518 nm for FITC, 410/455 nm for optical brighteners, and 540/625 nm for rhodamine.

### 2.12 Statistical analysis

Statistical analysis using SPSS (v27.0, SPSS Inc., USA). The mean is compared by the p < 0.05 least significant difference test. Graphing with GraphPad Prism 8.

## 3 Results and discussion

### 3.1 Fermentation ability analysis

**Fig. 1:**
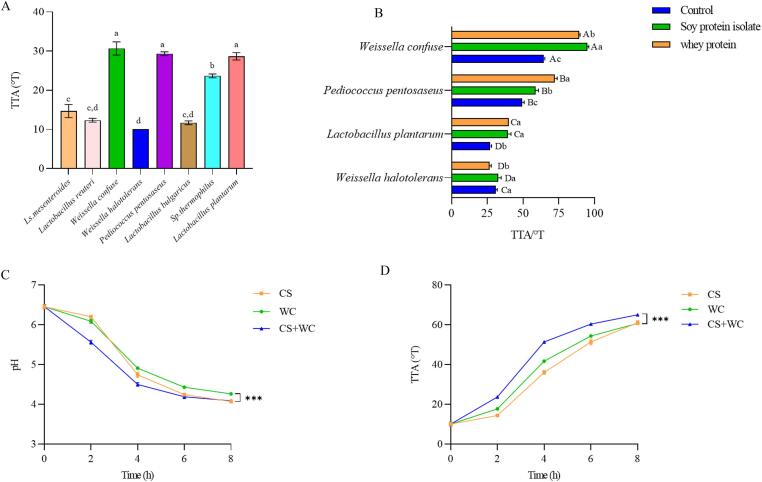
Comparison of different LAB and proteins and changes in pH (A) & (C) and TTA (B) & (D) during quinoa yogurt fermentation. *Note*: In Fig.1A, lowercase letters (a-f) represent significant differences in the TTA of fermented quinoa yogurt with different strains (p < 0.05). In Fig.1B, different lowercase letters indicate that the same bacteria added with different proteins (a-c: different proteins) have significant differences (p < 0.05); Different capital letters indicate that the same proteins are added to different fermented quinoa yogurt (A-D: different bacteria) with significant differences (p < 0.05).

As shown in Fig. 1A and Fig. S1A – C, the fermentation ability of different strains was compared. The pH, TTA, WHC, and TLC were used as indicators. Under the same fermentation conditions, the yogurt fermented with *W. confusa*, *L. plantarum,* and *P. pentosaceus* can produce lower pH and higher acidity. The yogurt fermented with *W. halophilus* showed the highest performance of WHC. All strains could reach 10^8^ CFU/g after fermentation, which was in line with the expectation of most researchers that the survival rate of probiotics remains above 10^6–7^ CFU/g during the shelf life (Zannini et al., 2018). The above four strains used in quinoa yogurt fermentation could maintain relatively high activity, indicating a better utilization of the nutrients in quinoa.

Nitrogen source is also an indispensable and important substance for LAB fermentation. The total protein content of quinoa is higher than that of rice, barley, corn, rye, and sorghum and is close to wheat, but it is still lower than ordinary milk powder. Our previous research found that the fermentation performance of quinoa yogurt was significantly improved by the addition of soy, which was rich in protein (Huang et al., 2022). Therefore, the effect of protein supplements on the fermentation performance of quinoa yogurt was explored by adding soybean protein and whey protein. As shown in Fig. 1B and Fig. S1D – F, the quinoa yogurt fermented with *W. confusa* presented the lowest pH and the highest level of TTA, indicating good substrate utilization. Compared with whey protein, soybean protein addition showed a better fermentation ability for *W. confusa*. Moreover, soybean protein utilization in quinoa yogurt would meet the demand for non-dairy development. In addition, previous studies have found that *W. confusa* could produce more exopolysaccharides when fermenting quinoa, showing potential in quinoa yogurt fermentation (Zannini et al., 2018). Therefore, *W. confusa* was selected and used in this study, combined with the commercial starter for quinoa yogurt development.

As shown in Fig. 1CD, fermentation kinetics were assessed and expressed according to the values of pH and TTA. At the beginning of fermentation (0–4 h), the modified commercial starter (CS + WC) exhibits more rapid fermentation characteristics than the commercial starter and *W. confusa*. Before 8 h, the fermentation rate of quinoa yogurt fermented with *W. confusa* (WC) was higher than that of commercial starter (CS). At 8 h, the TTA value of WC and CS was the same. The organic acids of quinoa yogurt fermented for 8 h were analyzed, as shown in Table S1. The increase of lactic acid, acetic acid, succinic acid, and citric acid was the main factor for the decrease in pH and the increase in TTA after fermentation. The fermentation rate of quinoa yogurt fermented with commercial starter and *W. confusa* kept staying at the highest level, with the highest content of lactic acid, acetic acid, succinic acid, and citric acid. Studies have shown that succinic acid is not only a substrate molecule for energy metabolism but also an important signaling molecule for important biological processes such as immunity, post-translational modification, inflammation, and tumors (Wan et al., 2020). All these results indicated that *W. confusa* could grow synergistically with *L. delbrueckii* and *S. thermophiles,* and the modified commercial starter could be applied in quinoa yogurt fermentation.

Our previous research demonstrated that soy is a suitable substrate for lactic acid bacteria (Huang et al., 2022). The proximate composition of quinoa yogurt before and after fermentation was determined (Table S2). With the addition of soy protein, the protein content of quinoa yogurt reaches 3 g/100 mL. Studies have shown that LAB can produce proteolytic enzymes and better utilize protein for life activities, which may be why LAB grows better in quinoa yogurt with added soy protein (Niccolai et al., 2020). Fermentation decreased carbohydrates, TSC, and calories and increased fat and protein content in yogurt. It indicates that LAB could secrete amylase and other carbohydrate enzymes during its growth. The content of fat and protein also increases correspondingly because the same amount of sample is taken during measurement.

### 3.2 Texture, rheological properties, and microstructure

**Fig. 2:**
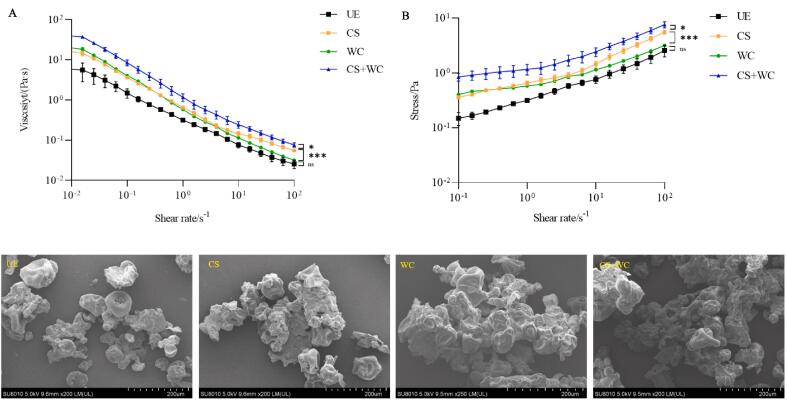
Effects of fermentation of different strains on rheological properties and microstructure of quinoa yogurt. (A) apparent viscosity; (B) Stress; (C) Scanning electron microscopy images.

The rheological properties of unfermented and fermented yogurt are shown in Fig. 2AB. The apparent viscosity of all samples decreases with the increase of shear rate, showing shear thinning. The stress increases with increasing shear rate and appears as a pseudoplastic liquid. At the beginning of the flow, pseudoplastic fluids exhibit a relatively high apparent viscosity due to their low shear rate. With the continuous increase of shear rate, the slope of the curve gradually decreases, and the viscosity of the liquid shows a downward trend (Li et al., 2021). CS + WC had the highest viscosity, mainly attributed to the production of exopolysaccharides. In previous studies, LAB exopolysaccharides have been shown to increase viscosity by forming network gel structures (Xu et al., 2018). The texture of the quinoa yogurt confirmed this result. As shown in Table S3. *W. confusa* can quickly use the substances in quinoa to produce organic acids in the early stage. It provided an acidic environment for the rapid growth of commercial starters, which enhanced the production of exopolysaccharides (EPS). The rheological property might be related to the microstructure of the sample, as shown in Fig. 2C. For the unfermented sample, discontinuous and broken starch granules were observed. However, a continuous and aggregated performance of the components was observed in the fermented sample. The aggregation might be attributed to the polysaccharides adhering to the surface of the protein and starch, which led to a stable and viscous network. This result was also confirmed by the change in shear stress; CS + WC also showed increased stress under the action of a strong gel network.

### 3.3 Storage stability evaluation

The physicochemical properties and the number of LAB of quinoa yogurt during storage were determined, as shown in Table S4. There was no change in pH and TTA during the 21-day storage of the three types of yogurts, indicating that quinoa yogurt may not have post-acidification behavior. Post-acidification is considered an undesirable process in yogurt products because it shortens shelf life and causes some defects, including severe acidity and dehydration shrinkage (Pachekrepapol et al., 2021). An important reason for post-acidification is that lactic acid bacteria continue using sugar in yogurt to produce lactic acid. However, quinoa yogurt contains only fewer sugars and has been depleted by lactic acid bacteria during the fermentation stage. In addition, a small amount of lactic acid bacteria activity during storage cannot play an enzymatic hydrolysis effect on starch and cannot provide the sugars required for post-acidification. After storage for 21 days, the TLC of each yogurt decreased during storage, and the ΔTLC of CS was the largest. The loss of LAB viability may be related to bacterial acid damage. The ΔTLC of CS + WC was the smallest, indicating that the synergistic fermentation of *W. confusa* and the commercial starter could improve the bacterial group vitality. On the other hand, soy protein contains carboxyl and amino groups, carboxyl groups can donate protons, and amino groups can accept protons. This conferred the strong buffering capacity of quinoa yogurt and reduced the damage of acid to bacteria. TLC of CS and WC decreased rapidly due to the reduction of available nutrients such as starch. Although the quantity decreased seriously, the final number of LAB concentrations could still reach more than 10^7^ CFU/mL.

WHC is an important indicator to judge the water-holding capacity of products and plays an important role in product taste analysis. As shown in Table s4, the WHC of all samples decreased significantly during storage. This is a common problem with yogurt products today, as lactic acid bacteria cannot produce enough EPS to maintain the yogurt network structure. Thickeners such as pectin are generally added in the industry to improve this result. Interestingly, WC had the lowest decline for the WHC. Previous studies have found that *W. confusa* can produce more EPS, improving the viscosity and water-holding capacity of quinoa yogurt (Zannini et al., 2018). While this is inconsistent with our conclusions, our study found that *W. confusa* appears to be good at maintaining the ability of quinoa yogurt to hold water during storage. Color is an important visual attribute of yogurt to enhance sensory evaluation. The L* of quinoa yogurt gradually increases during storage, which can increase the consumer's liking of the product. The decrease in the L* during yogurt storage due to proteolysis was not found in this study, which may be related to only including vegetable protein in yogurt (Campo et al., 2019). The a* showed a slight downward trend, and the b* showed a trend of rising first and then declining. These subtle changes may be related to lipid oxidation and Maillard reactions.

### 3.4 Total phenols and antioxidant activity

**Fig. 3:**
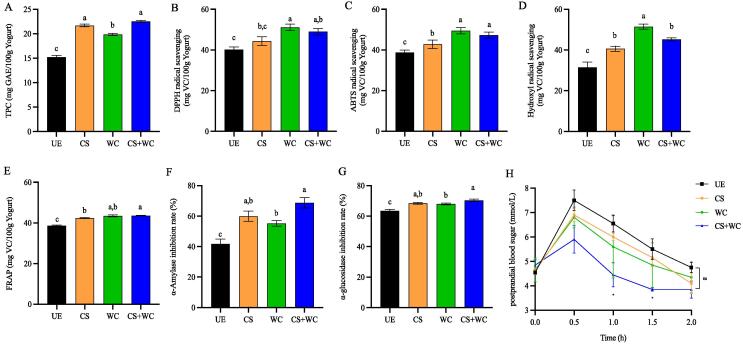
Effects of fermentation of different strains on quinoa yogurt total polyphenol content, antioxidant capacity, inhibition rate of digestive enzymes, and postprandial blood glucose in mice. (A) total phenolic content; (B) DPPH radical scavenging activity; (C) ABTS radical scavenging activity; (D) hydroxyl radical scavenging activity; (E) FRAP activity; (F) α-amylase inhibition; (G) α-glucosidase inhibition; (H) postprandial blood glucose in mice. *Note*: UE (unfermented sample), CS (quinoa yogurt fermented with commercial starter), WC (quinoa yogurt fermented with *W. confusa*), CS + WC (quinoa yogurt fermented with commercial starter and *W. confusa*); lowercase letters (a-f) represent fermentation of different strains of quinoa yogurt was significantly different (p < 0.05). In Fig 3. H, * represents the difference between UE and CS + WC (p < 0.1).

The polyphenols in quinoa yogurt were qualitatively and quantitatively, and the results are shown in Fig. 3A and Fig. S5. Compared to UE, fermentation significantly increased TPC. Quinoa was reported to be rich in phenolics, and the bound form was the dominant fraction (Zannini et al. 2018c). The hydrolase secreted by LAB fermentation acts on the plant cell wall, which can weaken the bond between the bound phenols and the cell wall, effectively releasing the bound polyphenols in the grains and converting them into soluble free polyphenols (Domínguez-Fernández et al., 2021). This is the main reason for the increase in TPC in quinoa yogurt. The highest TPC value of CS + WC reached 22.56 mg GAE/100 g, and epicatechin and p-hydroxybenzoic acid were the two most significant increase components, similar to the previous report (Melini and Melini 2021). The early acid production of *W. confusa* promotes the growth of commercial fermented strains, makes more TLC in yogurt, and promotes the release of bound phenols. However, the ability to release free polyphenols was not strong in the fermentation of *W. confusa* alone, indicating that combining *W. confusa* and a commercial starter is a good choice.

The antioxidant capacity of quinoa yogurt was assessed by DPPH, ABTS, hydroxyl radical scavenging activity, and FRAR, as shown in Fig. 3B – E. In line with the trend of polyphenols, fermentation can significantly improve the antioxidant capacity of quinoa yogurt. This is mainly attributed to LAB's ability to release free phenols. Interestingly, WC has the strongest antioxidant capacity, although the ability of *W. confusa* to release free polyphenols is not strong. This may be due to releasing one or several polyphenol monomers or carboxylic acids. Modified starter fermentation significantly improved the ABTS, hydroxyl radical scavenging ability, and FRAP of quinoa yogurt compared to commercial starter fermentation. Combined with the analysis in Table S5, it may be related to LAB to promote p-hydroxybenzoic acid and epicatechin release are involved. This suggests that the modified starter could significantly improve the antioxidant activity of quinoa yogurt, in which *W. confusa* played an important role.

### 3.5 Inhibits digestive enzymes and postprandial blood sugar

Diabetes mellitus is a metabolic disorder characterized by chronic hyperglycemia, and among the various treatments for diabetes, reducing postprandial hyperglycemia is the most important (Bilal Shah et al., 2018). According to reports, phenolic compounds are among the natural plant-based products that are useful for treating and preventing diabetes (Zhang et al., 2015). According to numerous studies, phenolic compounds restrict glucose absorption by preventing the activity of enzymes that break down carbohydrates, such as α-amylase and α-glucosidase (Zhang et al., 2015). Fig. 3F – H shows the evaluation and illustration of the inhibitory effects of quinoa yogurt on digestive enzymes and postprandial blood glucose in mice, including α-amylase and α-glucosidase. All samples' α-amylase inhibitory activity was significantly increased by fermentation, which is compatible with changes in polyphenol content. Studies have shown that proteolysis can block digestive enzymes and release polyphenols from phenol-protein complexes via LAB's secretion of proteolytic enzymes (Shori, 2020). Although the TPC of WC was lower than CS, the inhibitory activity of α-glucosidase was the same. The inhibition rate of α-amylase and α-glucosidase was greater than 70% in CS + WC, which exhibited the strongest inhibitory activity. The blood sugar levels of mice fed several varieties of quinoa yogurt were all greatest at 0.5 h after the meal. Only 5.8 mmol/L of blood glucose was found in mice fed CS + WC, 6.7 mmol/L in mice fed UE, and 7.5 mmol/L in mice fed CS. This was attributable to more LAB consuming more sugar during the quinoa yogurt's fermentation process. In addition, studies have shown that excessive accumulation of free radicals in the body is a potential molecular mechanism for inducing type II diabetes (Halim and Halim, 2019). However, CS + WC exhibits a strong free radical scavenging ability. On the other hand, blood glucose levels also directly relate to the activities of α-amylase and α-glucosidase. α-amylase and α-glucosidase can hydrolyze the α-1,4 and α-1,6 glycosidic bonds of starch to form oligosaccharides, resulting in increased blood glucose levels (Jang et al., 2018). However, CS + WC had the highest α-amylase and α-glucosidase inhibition rates.

### 3.6 Changes and correlation analysis of different components in simulated gastrointestinal digestion process

**Fig. 4:**
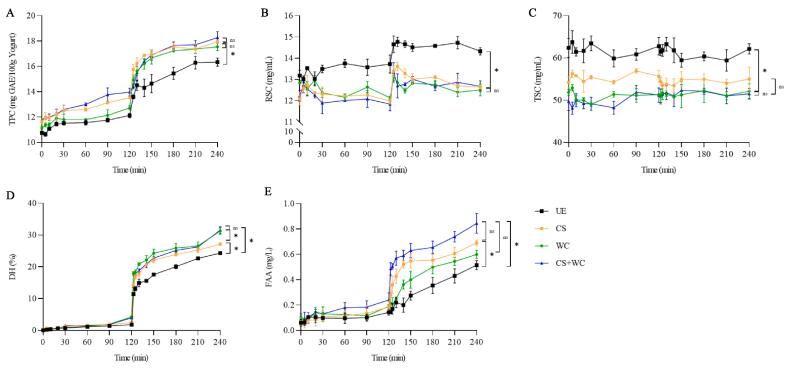
Changes of different components during simulated in vitro gastrointestinal digestion. (A) total phenolic content; (B) reducing sugar content; (C) total sugar content; (D) degree of protein hydrolysis; (E) the content of free fatty acid.

Changes in different components of quinoa yogurt during simulated in vitro digestion, including total polyphenol content (TPC), reducing sugar content (RSC), total sugar content (TSC), degree of protein hydrolysis (DH), and free fatty acid content (FAA) were evaluated and illustrated in Fig. 4. Gastrointestinal digestion increases TPC, suggesting that amylase, pepsin, trypsin, and a relatively low pH facilitate the release of free polyphenols from bound phenols. The same trend is found in yogurt with stevia water extract (Carvalho et al., 2019). Polyphenols were mainly released during intestinal digestion, and similar conclusions were found during in vitro digestion of grape polyphenols (Tagliazucchi et al., 2010). Antioxidant activities corroborated the results of TPC, including DPPH and ABTS free radical scavenging activities, as shown in Fig. S2. In contrast to gastric digestion, the antioxidant activity of quinoa yogurt that underwent intestinal digestion was significantly improved. Previous studies have shown that the radical scavenger activity of polyphenols has strongly pH-dependent (Tagliazucchi et al., 2010). The higher pH significantly increases this capability. The transition from the stomach to the intestinal environment can induce structural changes in phenolic molecules that can be attributed to the ionization of hydroxyl groups. CS + WC had the highest TPC and antioxidant activity during gastrointestinal digestion.

**Fig. 5:**
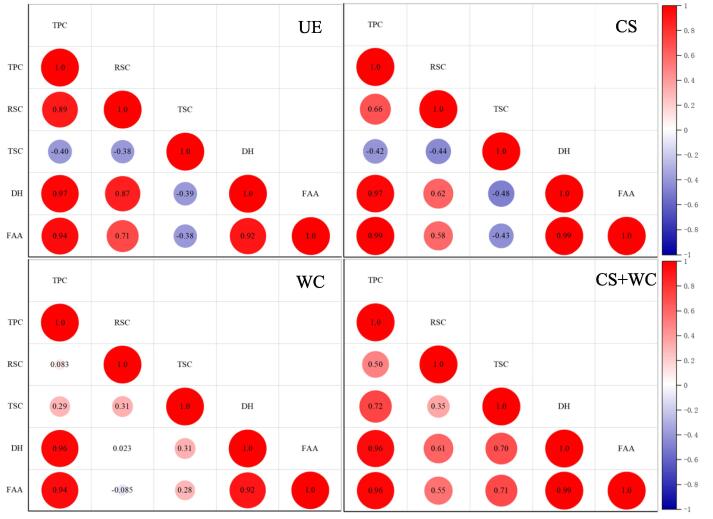
Correlation analysis of changes in protein hydrolysis degree, total sugar, reducing sugar content, total polyphenol content, and free fatty acid content during simulated in vitro gastrointestinal digestion. *Note*: TPC—total polyphenol content; RSC—reducing sugar content; TSC—total sugar content; DH/%—protein digestibility; FAA—free fatty acid release; UE (unfermented sample), CS (quinoa yogurt fermented with commercial starter), WC (quinoa yogurt fermented with *W. confusa*), CS + WC (quinoa yogurt fermented with commercial starter and *W. confusa*).

The analysis results of TPC, RSC, TSC, DH, and FAA correlation analysis of quinoa yogurt during simulated gastrointestinal digestion are shown in Fig. 5. The release of TPC, DH, and FAA was significantly positively correlated in all samples. CS + WC showed a stronger correlation among TPC, RSC, TSC, DH, and FAA during gastrointestinal digestion. This indicates that the decomposition of different components in the process of digestion can promote each other. On the other hand, the synergistic effect of *W. confusa* and commercial starter culture during fermentation promoted the pretreatment effect of quinoa yogurt by LAB. This is conducive to the human body's digestion and absorption of quinoa yogurt.

### 3.7 CLSM images of starch, fat, and protein in yogurt in vitro gastrointestinal digestion

The protein, fat, starch, and cellulose of quinoa yogurt were dyed with fluorescent dyes.

**Fig. 6:**
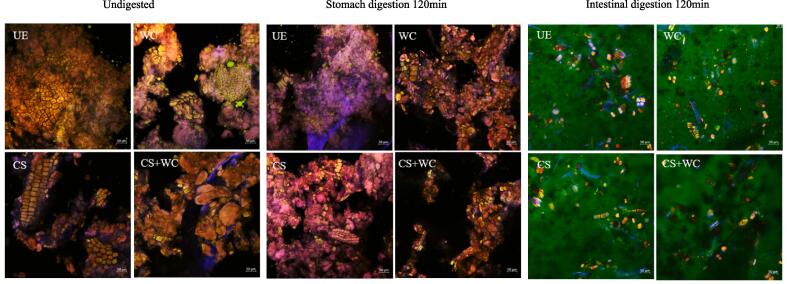
Changes in the microstructure of quinoa yogurt during simulated in vitro gastrointestinal digestion.

Changes in gastrointestinal digestion of starch, fat, and protein were observed using CLSM (Fig. 6). Compared with UE, *W. confusa* fermentation could decompose large fat globules into small fat particles. After gastric digestion, the fat morphology of UE did not change significantly, and the fat particles of quinoa yogurt were further decomposed. Similar changes were found in the gastrointestinal digestion of animal yogurt (Fguiri, 2017). The fat particles of CS + WC were more uniformly dispersed, which may be related to the synergistic effect of LAB fermentation on fat pretreatment and digestion of different components. Following intestinal digestion, the fat particles of the UE and quinoa yogurt disappeared, indicating that the fat was broken down into free fatty acids and other substances by pancreatic lipase.

After fermentation, the globular proteins of WC and CS + WC were exposed, while the proteins of UE and CS remained encapsulated by highly compacted plant tissue. After gastric digestion, the proteins of UE and CS are partially exposed, and the proteins of WC and CS + WC have been broken down by pepsin. Previous studies have shown that LAB fermentation can pretreat plant proteins and promote proteolysis during gastrointestinal digestion (Ketnawa et al., 2020). However, proteins tend to aggregate to form precipitates under acidic conditions, thereby preventing proteolysis. In addition, the trypsin-inhibitors present in soy protein, as a known 'antinutrient' component, can significantly compromise the protein digestibility. This results in a low level of protein digestion in the stomach. After intestinal digestion, there were no large protein particles in the UE and quinoa yogurt. In addition to the effect of proteases, the ability of polyphenols to improve protein digestion has also been reported in previous studies (Jakobek, 2015).

In contrast to the hydrolysis of proteins and fats. Before fermentation, most of the starch in quinoa has been decomposed into sugars by α-amylase and α-glucosidase for the life activities of LAB, and only a small amount of starch exists in plant tissues. There were closely arranged plant cells in UE, and the enzymes secreted by CS can only lose part of the plant cells. While *W. confusa* and the modified starter can lyse most of the closely arranged plant cells to release starch. The release of starch may be another important reason for the viscosity increase of CS + WC. It indicates that the enzymes secreted by LAB could break down plant cell walls, as found in a previous study (Nisa et al., 2019). The acidic environment of gastric digestion further promoted the decomposition of plant cells, and plant cells essentially disappeared in WC and CS + WC. After entering the intestine, under the action of pancreatic enzymes, the starch in the quinoa yogurt was broken down into sugars. As Fig. 4B shows, more reducing sugars were also produced. However, RSC decreased with increasing digestion time, possibly due to the oxidation of reducing sugars in an acidic environment (Shapla et al., 2018). WC and CS + WC had the lowest TSC, which may indicate that the biological activity of *W. confusa* is more dependent on sugar.

## 4 Conclusion

This study aimed to develop a novel functional quinoa yogurt with the modified commercial starter. The results showed that *W. confusa* had excellent fermentation performance of quinoa utilization. The modified commercial starter improved the fermentation rate of quinoa yogurt, and the highest TTA (70 °T) and organic acid contents were obtained after 8 h fermentation. The modified starter culture fermentation improved the texture and rheological properties of quinoa yogurt and formed a relatively compact microstructure (SEM). CS + WC had the lowest energy value (87 kal/200 mL) and the highest protein content (3 g/100 mL). During 21 days of storage, CS + WC showed more stable pH, TTA, TLC, and color than other yogurts. However, the WHC of CS + WC decreased significantly, which may be caused by the weakened protein gel network caused by the release of protease by LAB. The modified starter culture fermentation significantly increased the TPC, DPPH, ABTS, hydroxyl radical scavenging ability, and FRAP of quinoa yogurt. The inhibition rates of CS + WC on α-amylase and α-glucosidase were above 70%, which were significantly higher than those of other types of yogurts. Postprandial blood glucose levels in mice suggest that CS + WC has the potential to reduce postprandial blood glucose. The study also showed that CS + WC had a significant synergistic promoting effect on the digestion of polyphenols, proteins, and fats during in vitro gastrointestinal digestion (correlation coefficient > 0.9), which was verified by CLSM image and antioxidant activity. Therefore, it seems that CS + WC is a novel functional quinoa yogurt. The research will help in the development of quinoa fermentation products. In addition, more strains suitable for non-dairy fermentation should be screened to meet the development of plant-based fermented foods.

## Declaration of Competing Interest

The authors declare that they have no known competing financial interests or personal relationships that could have appeared to influence the work reported in this paper.

## Data Availability

Data will be made available on request.
