# Supplementary material for: Manufacture and characterization of a novel dairy-free quinoa yogurt fermented by modified commercial starter with Weissella confusa
Source: Food Chem X. 2023 Aug 6;19:100823. doi: 10.1016/j.fochx.2023.100823 (PMC10534153; doi:10.1016/j.fochx.2023.100823)
Supplement: Supplementary data 1 [file mmc1.docx]

**Supplementary figures**

Fig. S1. Screening of fermentation strains and proteins. (A) and (D) pH; (B) and (E) water holding capacity; (C) and (F) total lactic acid bacteria count


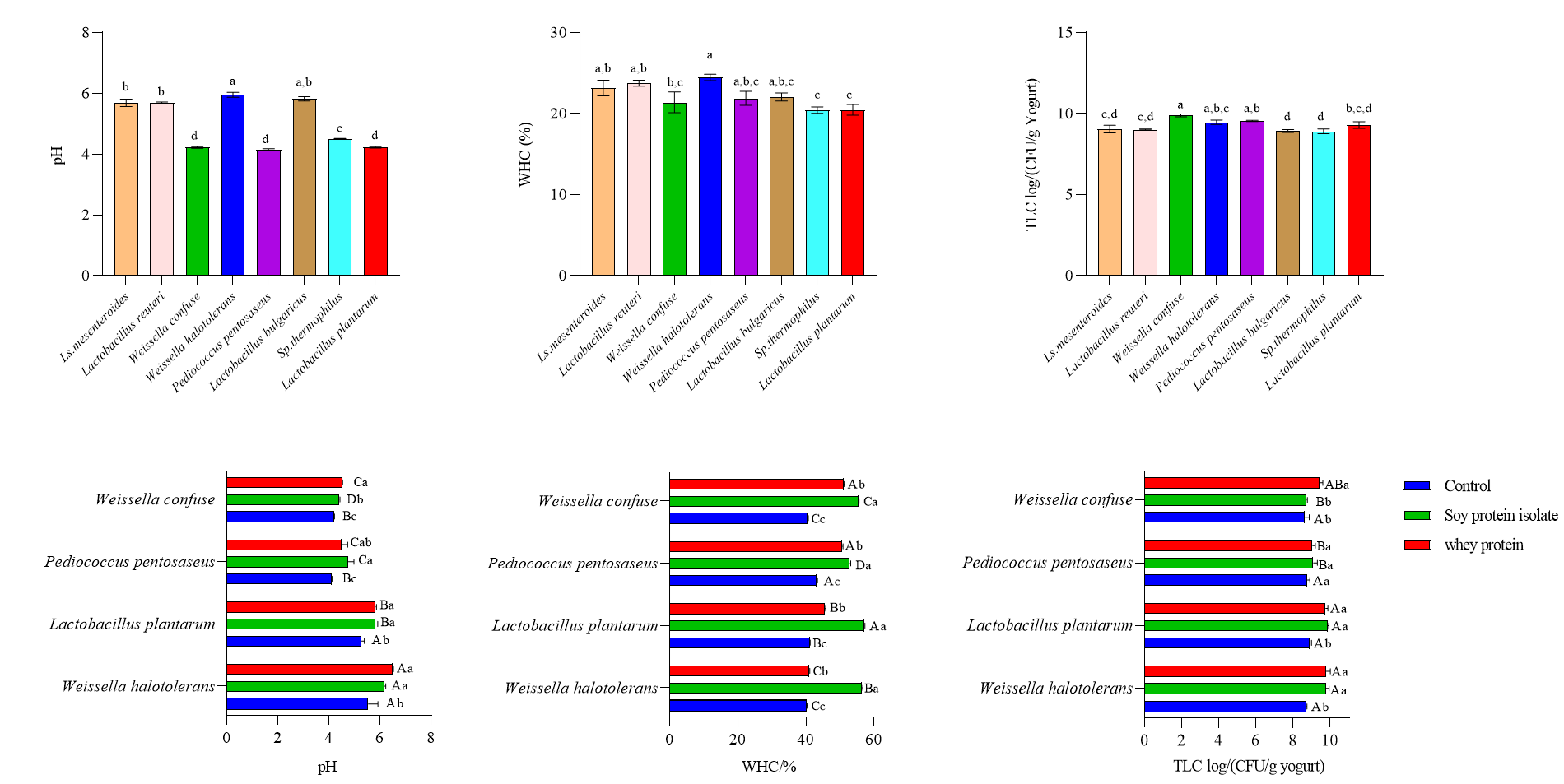


B

C

A

F

E

D

*Note*: Fig.1A-C, pH, TTA, and TLC of quinoa yogurt fermented with different strains. Lowercase letters (a-d) represent significant differences in quinoa yogurt fermented with different strains.

Fig.1D-F, pH, TTA, and TLC of quinoa yogurt fermented with different proteins and different strains. Different lowercase letters indicate that the same bacteria added with different proteins (a-c: different proteins) have significant differences (p < 0.05); Different capital letters indicate that the same proteins are added to different fermented quinoa yogurt (A-D: different bacteria) with significant differences (p < 0.05).

Fig. S2. Changes in the antioxidant capacity of quinoa yogurt during simulated in *vitro* gastrointestinal digestion. (A) DPPH radical scavenging activity; (B) ABTS radical scavenging activity.

A

B

*Note*: UE (unfermented sample), CS (quinoa yogurt fermented with commercial starter), WC (quinoa yogurt fermented with *W. confusa*), CS+WC (quinoa yogurt fermented with commercial starter and *W. confusa*); Capital letters (A-C) indicate that the same type of yogurt in different digestion times have significant differences (p < 0.05), and lowercase letters (a-c) indicate that the same digestion times for different types of yogurt have significant differences (p < 0.05).
