# Supplementary material for: Manufacture and characterization of a novel dairy-free quinoa yogurt fermented by modified commercial starter with Weissella confusa
Source: Food Chem X. 2023 Aug 6;19:100823. doi: 10.1016/j.fochx.2023.100823 (PMC10534153; doi:10.1016/j.fochx.2023.100823)
Supplement: Supplementary data 2 [file mmc2.docx]

Supplemental tables

Table S1. Types and contents of organic acids in fermented quinoa yogurt with different strains

| Composition | UE | CS | WC | CS+WC |
| --- | --- | --- | --- | --- |
| Tartaric Acid | 6.77±0.43^a^ | 6.20±0.27^a^ | 8.00±0.39^a^ | 6.73±0.15^a^ |
| Oxalic Acid | 0.76±0.11^a^ | 0.86±0.07^a^ | 0.84±0.05^a^ | 1.02±0.02^a^ |
| Malic Acid | 10.25±1.16^a^ | 9.65±1.21^a^ | 8.27±0.91^a^ | 11.27±0.37^a^ |
| Formic Acid | 8.00±0.91^a^ | 7.53±0.94^a^ | 6.45±0.71^a^ | 9.02±0.52^a^ |
| Lactic Acid | 0.00±0.00^b^ | 6.98±0.34^a^ | 8.07±0.51^a^ | 7.50±0.17^a^ |
| Acetic Acid | 0.00±0.00^b^ | 7.68±0.37^a^ | 8.88±0.56^a^ | 8.25±0.18^a^ |
| Citric Acid | 0.00±0.00^b^ | 5.00±0.81^a^ | 3.73±0.58^a^ | 4.19±0.11^a^ |
| Succinic Acid | 0.00±0.00^b^ | 5.46±1.24^a^ | 2.47±0.68^ab^ | 6.48±0.20^a^ |
| Propionic Acid | 0.43±0.36^a^ | 0.01±0.01^a^ | 0.00±0.00^a^ | 0.53±0.10^a^ |
| Fumaric Acid | 0.35±0.06^a^ | 0.11±0.02^b^ | 0.17±0.02^ab^ | 0.14±0.01^b^ |
| Butyric Acid | 0.00±0.00^d^ | 0.62±0.02^b^ | 1.72±0.01^a^ | 0.28±0.01^c^ |

Results are presented as mean ± S.E.M (n=3). Different lowercase letters (a-d) represent significant differences (p < 0.05) in the organic acid content (mg/mL) of quinoa yogurt. UE (unfermented sample), CS (quinoa yogurt fermented with commercial starter), WC (quinoa yogurt fermented with *W. confusa*), CS+WC (quinoa yogurt fermented with commercial starter and *W. confusa*).

Table S2. Effects of fermentation of different strains on basic components of quinoa yogurt

| Composition | UE | CS | WC | CS+WC |
| --- | --- | --- | --- | --- |
| Ash（%） | 0.29±0.01^c^ | 0.33±0.01^bc^ | 0.36±0.01^b^ | 0.41±0.01^a^ |
| Protein（%） | 2.73±0.03^b^ | 3.05±0.05^a^ | 2.77±0.06^ab^ | 3.00±0.05^ab^ |
| Fat（%） | 0.32±0.00^b^ | 0.36±0.02^ab^ | 0.39±0.01^a^ | 0.38±0.01^ab^ |
| Total solids（%） | 11.05±0.02^a^ | 10.57±0.05^ab^ | 10.45±0.02^c^ | 10.55±0.04^bc^ |
| Carbohydrate（%） | 7.70±0.02^a^ | 6.83±0.00^b^ | 6.94±0.05^b^ | 6.75±0.12^b^ |
| Moisture（%） | 88.95±0.02^c^ | 89.43±0.05^b^ | 89.55±0.02^a^ | 89.45±0.04^ab^ |
| Kcal（200 mL） | 91.48±0.11^a^ | 87.60±0.75^b^ | 86.91±0.15^b^ | 87.06±0.32^b^ |

Results are presented as mean ± S.E.M (n=3). Different lowercase letters (a-c) represent significant differences in the basic components of different types of fermented quinoa yogurt (p < 0.05). UE (unfermented sample), CS (quinoa yogurt fermented with commercial starter), WC (quinoa yogurt fermented with *W. confusa*), CS+WC (quinoa yogurt fermented with commercial starter and *W. confusa*).

Table S3. Effects of fermentation of different strains on the texture properties of quinoa

| Items | Hardness (g) | Adhesiveness (g·s) | Chewiness | Gumminess | Springiness | Cohesiveness |
| --- | --- | --- | --- | --- | --- | --- |
| CS | 44.18±0.83^ab^ | 0.00±0.00^a^ | 41.45±0.47^ab^ | 43.23±0.24^ab^ | 096±0.01^a^ | 0.99±0.01^a^ |
| WC | 43.88±0.23^b^ | 0.00±0.00^a^ | 40.98±0.30^b^ | 42.87±0.43^b^ | 0.95±0.00^ab^ | 0.98±0.01^a^ |
| CS+WC | 44.81±0.46^a^ | 0.00±0.00^a^ | 41.55±0.89^a^ | 43.80±0.67^a^ | 0.95±0.00^b^ | 0.98±0.01^a^ |

Results are presented as mean ± S.E.M (n=6). Different lowercase letters (a-c) represent significant differences in the texture properties of quinoa yogurt (p < 0.05). CS (quinoa yogurt fermented with commercial starter), WC (quinoa yogurt fermented with *W. confusa*), CS+WC (quinoa yogurt fermented with commercial starter and *W. confusa*).

Table S4. Changes in physicochemical properties and number of lactic acid bacteria during storage of quinoa yogurt

| Items | Refrigerated storage | CS | WC | CS+WC |
| --- | --- | --- | --- | --- |
| pH | Day 1 | 3.97±0.04^ABa^ | 4.05±0.06^Aa^ | 3.83±0.03^Cb^ |
|  | Day 7 | 4.06±0.04^ABa^ | 4.15±0.05^Aa^ | 3.90±0.03^BCb^ |
|  | Day 14 | 4.09±0.06^Ab^ | 4.23±0.03^Aa^ | 4.02±0.02^ABb^ |
|  | Day 21 | 3.93±0.03^Cb^ | 4.11±0.07^Aa^ | 4.01±0.04^Aab^ |
| Titratable Acidity (°T） | Day 1 | 74±1.25^Ab^ | 78±0.82^Aa^ | 80±0.47^BCa^ |
|  | Day 7 | 73±0.82^Ab^ | 77±1.25^Aa^ | 78±0.82^Ca^ |
|  | Day 14 | 74±1.25^Ab^ | 80±0.47^Aa^ | 83±0.47^Aa^ |
|  | Day 21 | 75±0.82^Ab^ | 78±1.63^Aab^ | 81±0.47^Ba^ |
| TLC (CFU/g) | Day 1 | 4.30×10^10^±0.05^Aa^ | 2.19×10^10^±0.06^Aa^ | 3.10×10^10^±0.15^Aa^ |
|  | Day 7 | 4.30×10^9^±0.03^Ba^ | 9.47×10^9^±0.22^Ba^ | 8.53×10^9^±0.10^Ba^ |
|  | Day 14 | 3.60×10^8^±0.16^Cb^ | 8.47×10^8^±0.07^Ba^ | 9.27×10^8^±0.05^Ba^ |
|  | Day 21 | 3.80×10^7^±0.08^Cb^ | 1.46×10^7^±0.05^Bb^ | 9.07×10^7^±0.12^Ba^ |
| WHC (%) | Day 1 | 31.33±0.60^Aa^ | 31.78±0.74^Aa^ | 31.98±0.61^Aa^ |
|  | Day 7 | 31.62±0.06^Aa^ | 31.10±0.40^ABa^ | 30.52±0.74^ABa^ |
|  | Day 14 | 30.93±0.95^Aa^ | 30.47±0.93^ABa^ | 29.40±0.11^Ba^ |
|  | Day 21 | 26.45±0.45^Bb^ | 29.05±0.65^Ba^ | 25.92±0.74^Cb^ |
| L* | Day 1 | 25.03±0.04^Ca^ | 23.88±0.11^Cb^ | 25.25±0.29^Ba^ |
|  | Day 7 | 26.30±0.44^Bb^ | 26.41±0.07^Bb^ | 28.05±0.49^Aa^ |
|  | Day 14 | 28.54±0.06^Aa^ | 28.18±0.39^Aa^ | 28.29±0.60^Aa^ |
|  | Day 21 | 27.88±0.22^Aa^ | 26.68±0.24^Bb^ | 27.62±0.27^Aa^ |
| a* | Day 1 | 0.37±0.03^Ab^ | 0.32±0.02^Ab^ | 0.48±0.33^Aa^ |
|  | Day 7 | 0.24±0.01^Bab^ | 0.26±0.00^Ba^ | 0.21±0.01^Bb^ |
|  | Day 14 | 0.36±0.03^Aa^ | 0.27±0.03^ABa^ | 0.26±0.06^Ba^ |
|  | Day 21 | 0.19±0.01^Bab^ | 0.24±0.00^Ba^ | 0.14±0.04^Bb^ |
| b* | Day 1 | 4.90±0.11^Ba^ | 4.54±0.03^Cb^ | 5.01±0.13^ABa^ |
|  | Day 7 | 4.90±0.04^Bb^ | 5.04±0.13^ABab^ | 5.18±0.06^Aa^ |
|  | Day 14 | 5.43±0.11^Aa^ | 5.17±0.09^Aa^ | 5.25±0.06^Aa^ |
|  | Day 21 | 5.09±0.15^ABa^ | 4.86±0.04^Ba^ | 4.88±0.01^Ba^ |

Results are presented as mean ± S.E.M (n=3). The means of different capital letters (A-C: refrigeration time) of the same yogurt type were significantly different (p < 0.05). Means of different lowercase letters (a-d: yogurt type) on the same day were significantly different (p < 0.05). CS (quinoa yogurt fermented with commercial starter), WC (quinoa yogurt fermented with *W. confusa*), CS+WC (quinoa yogurt fermented with commercial starter and *W. confusa*).

Table S5. Analysis of main components of free polyphenols in quinoa yoghurt fermented by different strains

| Composition | UE | CS | WC | CS+WC |
| --- | --- | --- | --- | --- |
| Gallic acid | 1.63±0.04^a^ | 1.06±0.02^b^ | 0.98±0.01^b^ | 1.05±0.10^b^ |
| 3,4-Dihydroxybenzoic acid | 4.95±0.01^a^ | 4.16±0.08^c^ | 4.88±0.01^a^ | 4.62±0.01^b^ |
| Neochlorogenic acid | 2.09±0.03^a^ | 2.03±0.04^a^ | 2.19±0.01^a^ | 2.13±0.06^a^ |
| Epigallocatechin | 0.00±0.00^a^ | 0.00±0.00^a^ | 0.00±0.00^a^ | 0.00±0.00^a^ |
| p-hydroxybenzoic acid | 1.48±0.02^b^ | 1.65±0.02^a^ | 1.53±0.01^b^ | 1.64±0.00^a^ |
| Catechin | 0.63±0.07^a^ | 0.71±0.06^a^ | 0.61±0.03^a^ | 0.71±0.04^a^ |
| Chlorogenic acid | 1.68±0.57^a^ | 1.70±0.10^a^ | 1.60±0.02^a^ | 1.56±0.03^a^ |
| Epicatechin | 0.57±0.03^b^ | 0.70±0.03^ab^ | 0.75±0.01^a^ | 0.68±0.03^ab^ |
| Quercetin | 31.04±0.22^a^ | 28.67±1.88^a^ | 30.58±0.55^a^ | 29.30±0.34^a^ |

Results are presented as mean ± standard error (n=3). Different lowercase letters (a-c) represent significant differences (p < 0.05) in the polyphenol content (μg/mL) of quinoa yogurt. UE (unfermented sample), CS (quinoa yogurt fermented with commercial starter), WC (quinoa yogurt fermented with *W. confusa*), CS+WC (quinoa yogurt fermented with commercial starter and *W. confusa*).
